# Women’s perception of support and control during childbirth in The Gambia, a quantitative study on dignified facility-based intrapartum care

**DOI:** 10.1186/s12884-018-2025-5

**Published:** 2018-10-23

**Authors:** Saffie Colley, Chien-Huei Kao, Meeiling Gau, Su-Fen Cheng

**Affiliations:** 1 Ministry of Health & Social Welfare, West Africa Banjul, The Gambia; 2 Graduate Institute of Nurse-Midwifery National Taipei University and Health Sciences, 365 Ming-Te Road, Taipei, 112 Taiwan; 3 Graduate Institute of Nursing, National Taipei University and Health Sciences, 365 Ming-Te Road, Taipei, 112 Taiwan

**Keywords:** Perception, Control, Support, Childbirth, Women

## Abstract

**Background:**

In The Gambia, a woman faces 1 in 24-lifetime risk of maternal death due to pregnancy and childbirth, yet, only 57% of deliveries are conducted by skilled birth attendants. However, poor provider attitude has been identified as one of the contributing factors hampering the efforts of the government in improving access to skilled care during childbirth. This study, therefore, explored women’s perception of support and control during childbirth in The Gambia.

**Methods:**

A descriptive cross-sectional study was employed. A convenience sampling method was used to select participants in two regions in The Gambia. A sample size of 200 women who met the eligibility criteria was recruited after informed consent. The demographic-obstetric information sheet and the Support and Control in Birth scale (SCIB) were used to collect data. Data analysis was done using SPSS software version 23.0.

**Results:**

Women’s perceptions of support and control were low. External control 1.85 (SD ± 0.43) recorded the least perception compared to internal control 2.41 (SD ± 0.65) and perception of support 2.52 (SD ± 0.61). Participants reported the lowest perceptions in pain control, involvement in decision making, information sharing and the utilization of different position during birth. Women’s age (*p* < .001) and mode of delivery (*p* = .01), significantly predicted women’s perception of internal control. Educational status (*p* = .02), mode of delivery (*p* = .04), place of delivery (*p* < .001) and perception of support (*p* < .001) significantly predicted women’s perception of external control, whilst birth plan (*p* = .001), mode of delivery (*p* = .04), and perception of external control (*p* < .001) significantly predicted women’s perception of support.

**Conclusion:**

This study concluded that an environment that promotes women feeling a sense of control and support during childbirth should be created in order to ensure a dignified intrapartum care in The Gambia. This can be achieved through effective training of skilled birth attendants on non-pharmacological pain management, effective communication with clients and promoting women’s participation in decision-making regarding their care throughout the process of childbirth.

**Electronic supplementary material:**

The online version of this article (10.1186/s12884-018-2025-5) contains supplementary material, which is available to authorized users.

## Background

In The Gambia, a woman faces 1 in 24-lifetime risk of maternal death due to pregnancy and childbirth, yet, only 57% of deliveries are conducted by skilled birth attendants [1]. Ensuring universal access to reproductive health service is the aim of all health systems in developing countries including The Gambia, in an effort to reduce maternal morbidity and mortality so as to meet the Sustainable Development Goals by 2030. One of the strategies in achieving this aim is the promotion of skilled care before, during and after childbirth [2]. However, poor provider attitude has been identified as one of the contributing factors hampering the efforts of The Gambian government in improving access to skilled birth care [3]. Addressing the key obstacles to the utilization of skilled care and creating an environment that supports women’s needs during childbirth is, therefore, crucial [4].

Childbirth is a stressful experience for women worldwide, therefore, it is of paramount importance for caregivers to be supportive and create an atmosphere that allows women to gain autonomy over the process of childbirth to ensure positive dignified birth experience [2, 5]. Ensuring client satisfaction, promoting clients participation in decision making about their care, and providing a respectful and nondiscriminatory health care are among The Gambia national health policy guiding principles, to improving access to quality healthcare delivery [6]. Supportive care from providers and care that help women to obtain their level of control enhances dignity during childbirth [7], promote women’s participation in decision-making regarding their care [8], reduce obstetric interventions during birth [9, 10], and promote positive decisions concerning the utilization of maternity health services in future pregnancies [11]. Women’s loss of control and lack of supportive care are forms of mistreatment during childbirth and may have an effect on decisions about the utilization of intrapartum care services in the future [11].

Studies on factors influencing the utilization of maternal healthcare services in The Gambia mainly focused on economic and sociocultural factors [12, 13]. Little attention is given to understanding women’s view about intrapartum care services that recognize their needs. This study, therefore, seeks to assess women’s perception of support and control during childbirth in The Gambia. The findings will help midwives to have a better understanding of women’s needs during intrapartum care to enhance effective dignified maternity care. The findings will also help to address the challenges faced by developing countries of moving towards dignified childbirth.

The aim of this study was to explore women’s perception of support and control during childbirth in The Gambia and to identify related factors influencing perceptions of support and control during childbirth. The findings will not only help to improve obstetric care in The Gambia but, will contribute to the global body of knowledge related to dignified maternity care.

## Methods

### Setting and sample

A quantitative, cross-sectional descriptive approach was employed. Due to the limited time available and the lack of funding to conduct this study, convenience sampling was used to select the study areas and recruit participants, between August 2016 and September 2016, at three major health centers in Western and Lower River Regions in The Gambia. Western Region is located in the western part of The Gambia, with nearly 75% of the total population [14] and the highest number of deliveries (approximately 27,200 per year) [15]. Lower River Region which is in the southern part of the country has a population of 82,361 [14] and a number of about 2000 deliveries annually [15].

Based on the two stated objectives to this paper; (1) to assess women’s perception of support and control during childbirth; (2) to identify related factors influencing perceptions of support and control during childbirth, the researchers calculated a sample size that will achieve both the descriptive and inferential statistics needed to analyze the data. The researchers used a 95% confidence level, an estimated number of deliveries of 29,200 per year in Western and Lower River Regions [14, 15], an estimated confidence interval of 10% from a previous study on a similar population [16] and a 10% non-response rate. The minimum sample size required for the descriptive statistics was 165 participants. The G-power 3.1.9.2 was used to calculate the sample size for the inferential statistics using an F test, a linear multiple regression with fixed model and R^2^ increase, effect size of 0.2, alpha of 0.05, a power (1-β err prob) of 0.95, 9 predictors (all tested), and a 10% non-response rate. A minimum sample size of 140 participants was required. However, we recruited 200 participants in this study to ensure that all statistical analysis for the two research objectives required were achieved.

The predictors used in this study included the independent variables (demographic and obstetric characteristics of participants). The operational definitions of these predictors are clearly stated in the research instrument section in this paper.

### Eligibility criteria for participants

Women between the ages of 18 and 35 years, with no medical or obstetric complication during pregnancy, were eligible to participate. They were also eligible if admitted for at least 3 h in a public major health facility in Western and Lower River Regions prior to delivery. Women with multiple pregnancies and those who delivered before arrival at the health facility were excluded.

### Data collection process and instruments

Ethics approval was obtained from The Gambia government and Medical Research Council ethics committee, reference number R016005. A demographic-obstetric questionnaire (Additional file 1) and the Support and Control in Birth (SCIB) scale (Additional file 2) were used to obtain participants’ information. The structured demographic-obstetric questionnaire was developed by the researcher based on evidence. That is, the variables that were included in the questionnaire such as age, education, place of delivery, marital status, parity, number of antenatal attendance and birth plan were selected based on similar studies [17–22]. Participants in this study were classified as either married or single. The married participant was classified as being in monogamous or polygamous families, and those not married were single, cohabitating, or widowed. Age of participants ranges from 18 to 35 years, participants ages were grouped as 24 years and below and 25 years and above. This age cut-off was based on a previous study by Aitken and colleagues [23]. Educated participants were those with primary education or above, and those uneducated have no formal education. The parity of participants’ ranges from 1 to 4, primiparous were participants who have given birth for the first time and multiparous were those having given birth two or more times. Birth planning is one of the components of antenatal care in The Gambia and in this study, a participant who had a birth plan is one who was educated on a birth preparedness plan and complication readiness in one or more antenatal visits during pregnancy. Participants ethnicities in this study are those found in The Gambia [14].

The Support and Control in Birth (SCIB) scale was developed by Ford et al. [22]. It is a 33-item scale with three subscales, namely; internal control, external control, and support. Internal control subscale assessed participants’ ability to control oneself during childbirth including events such as pain, emotion, behavior, physical functioning and thoughts through their own efforts. The external control subscale assessed participants’ control over events being controlled by external forces such as pain relief (comfort measures or analgesic), information, environment, decisions and procedures, and birth outcome. The support subscale assessed coaching, coping techniques, staff attitude, empathy, understanding and reassurance, encouragement, listening to women’s wishes, informational support, and physical support for pain relief.

The SCIB scale is a 5-point Likert scale with responses ranging from completely agree to completely disagree, the middle number (3) representing neither agree nor disagree. The 5-point response scale ranges from 1 to 5, with high scores indicating more support or control, low scores indicating less support or control, and a score of 3 indicating an average support or control. Negative items in the scale were reversed scored. The scale has an overall Cronbach’s alpha coefficient of 0.95, internal subscale (0.86), external subscale (0.93) and the support subscale (0.93) [22]. It was tested by Ford et al. (2009) and the psychometric properties were compared well with other published scale of the same field, notably, the Labour Agentry Scale [22]. Similarly, the scale was used by Inci, Gokce, and Tanhan (2015), to develop and validate a Turkish version of the SCIB scale, which was concluded to be reliable and valid with an internal consistency coefficient of 0.86 [24]. All the variables explained in the subscales are incorporated in the health system of the Gambia as per WHO standard. However, as a result of the difference in demography and culture, the SCIB scale was adapted and it was pretested to ensure consistency. The Cronbach’s alpha for the total scale was 0.78, internal control subscale was 0.78, external control subscale was 0.75 and support subscale was 0.81. The Cronbach’s alpha in this study is lower compared to a study conducted in the United Kingdom, to measure maternal perception of support and control during childbirth [22]. The inter-items correlation indicated an increase in the Cronbach’s Alpha if items such as “I was overcome by the pain” and “I gained control by working with my body” in the internal control subscale and “I could get up and move around as much as I wanted” in the external control subscale, were deleted. There was no irrelevant item in the support subscale. Cultural beliefs and demography of participants may be responsible for the difference in the Cronbach’s Alpha of the two studies.

In each health facility, participants admitted more than 3 h post- delivery in the postnatal ward for observation, and who met the eligibility criteria and signed the consent form, were invited to participate. As a result of the low literacy rate among women in The Gambia, the questionnaires were administered to the eligible participants that were in the ward at the time of data collection and are willing to participate using the face-to-face interview.

### Data processing and analysis

SPSS statistical software version 23.0 was used to analyze the data. Frequency distributions, mean and standard deviation were used to analyze participants’ demographic-obstetric characteristics and perceptions of support and control during childbirth. As a result of the non-normal data, non-parametric tests such as Mann-Whitney *U* test, Kruskal-Wallis *H* test and Spearman’s *rho* correlation were used to compare difference in participants’ demographic-obstetric characteristics and perceptions of support and control during childbirth, and relationship between perceptions of support and control during childbirth. Linear multiple regression was used to analyze participants’ demographic-obstetric characteristics predicting perceptions of support and control during childbirth.

## Results

### Demographic and obstetric characteristics of study participants

In this study, there was a 100% response rate and no missing data. Most (55%, *n* = 110) of the participants had no formal education, were married (97%, *n* = 194), and of Mandinka ethnicity (40.0%, *n* = 80). The mean age of the participants was 25. 8 (SD ± 5.09), and about three quarters (74%, *n* = 148) were multiparous. More than half (50.5%, *n* = 101) of the participants had less than four antenatal visits, reported having a birth plan (55.5%, *n* = 111) and had a normal vaginal delivery (95.5%, *n* = 191). In terms of place of delivery, majority of participants delivered in Western Two Health Region (37.5%, *n* = 75). Table 1 shows the demographic and obstetric characteristics of participants.

**Table 1:** Demographic-obstetric Characteristic of Participants (*N* = 200)

Variables	n (%)	Mean ± SD	Range
Age		25.82 ± 5.09	18–35 Yrs.
Less than 25 yrs	83 (41.5)		
25 yrs. or more	117 (58.5)		
Education			
No formal education	110 (55.0)		
Primary education and above	90 (45.0)		
Marital status			
Married (monogamy or polygamy)	194 (97.0)		
Not married (single, cohabiting, widowed)	6 (3.0)		
Ethnicity			
Jola	18 (9.0)		
Fula	47 (23.5)		
Wollof	32 (16.0)		
Mandinka	80 (40.0)		
Others	23 (11.5)		
Parity			1–4
Nullipara (1)	52 (26.0)		
Multipara (≥ 2)	148 (74.0)		
No. of antenatal visits			
Less than 4	101 (50.5)		
4 or more	99 (49.5)		
Place of delivery			
Western one health region	70 (35.0)		
Western two health region	75 (37.5)		
Lower river health region	55 (27.5)		
Birth plan			
No	89 (44.5)		
Yes	111 (55.5)		
Mode of Delivery			
Vaginal delivery	191 (95.5)		
Assisted vacuum delivery	3 (1.5)		
Caesarean section	6 (3.0)		

### Women’s perception of support and control during childbirth

#### Perception of internal control

The mean score for internal control was 2.41 (SD ± 0.65), indicating a low perception. The three lowest mean scores in this subscale included; “I was able to control my reactions to the pain” 2.03 (± 1.00); “I was mentally calm” 2.07 (SD ± 1.17); and “the pain was too great for me to gain control over it” 2.09 (SD ±1.08). Items with the highest scores were “I was overcome by the pain” 3.76 (SD ± 0.97); and “I gained control by working with my body” 3.29 (SD ± 1.19).

#### Perception of external control

External control, 1.85 (SD ± 0.43) equally revealed a low perception. Of the 11 items, the three lowest scores were observed in, “I could decide when I received information” 1.48 (SD ± 0.56); “I could influence which procedures were carried” 1.54 (SD ± 0.61); and “I had control over what information I was given” 1.56 (SD ± 0.63). The only item with a high mean score was observed in “I could get up and move around as much as I wanted” 3.59 (SD ± 1.32).

#### Perception of support

The overall mean score for support was 2.52 (SD ± 0.62), revealing a moderately low perception and the items such as “the staff helped me to try different positions” 1.45 (SD ± 0.89); “I was given time to ask questions” 2.01 (SD ± 1.24); and “the staff stopped doing something if I asked them to stop” 2.18 (SD ± 1.03), showed the lowest mean scores. Items with high scores were “the staff realized the pain I was in” 3.45 (SD ± 1.17); the staff encouraged me not to fight what my body was doing” 3.20 (SD ± 1.15); and “the staff helped me find energy to continue when I wanted to give up” 2.98 (SD ± 1.29). Table 2 shows women’s perception of maternal support and control during childbirth.

**Table 2:** Women’s Perception of Support and Control during Childbirth (*N* = 200) [22]

Variables	Mean ± SD	Ranking	
		Lowest	Highest
Internal control	2.41 ± 0.65		
1.The pain was too great for me to gain control over it*	2.09 ± 1.08	3	
2.I was overcome by the pain	3.76 ± 0.97		1
3.I was able to control my reactions to the pain	2.03 ± 1.00	1	
4.I was mentally calm	2.07 ± 1.17	2	
5.I was in control of my emotions	2.22 ± 1.21		
6.I felt my body was on a mission that I could not control*	2.16 ± 1.18		
7.Negative feelings overwhelmed me*	2.16 ± 1.21		
8.I gained control by working with my body	3.29 ± 1.19		2
9.I could control the sounds I was making	2.15 ± 1.14		
10.I behaved in a way, not myself*	2.18 ± 1.13		
External control	1.85 ± 0.43		
1. I had control over when procedures happened	1.57 ± 0.64		
2.I could influence which procedures were carried	1.54 ± 0.61	2	
3.I decided whether procedures were carried out or not	1.61 ± 0.73		
4.People in the room took control*	1.94 ± 0.80		
5.I had control over the decisions that were carried out or not	1.75 ± 0.84		
6.I could get up and move around as much as I wanted	3.59 ± 1.32		1
7.People coming in and out of the room was beyond my control*	1.92 ± 1.12		
8.I chose whether I was given information or not	1.54 ± 0.68	3	
9.I could decide when I received information	1.48 ± 0.56	1	
10.I had control over what information I was given	1.56 ± 0.63		
11. I felt I had control over the way my baby was finally born	1.90 ± 0.70		
Support	2.52 ± 0.62		
1. The staff helped me find the energy to continue when I wanted to give up.	2.98 ± 1.29		3
2. The staff seemed to know instinctively what I wanted or needed	2.68 ± 1.15		
3. The staff went out of their way to try a new way to try to keep me comfortable.	2.73 ± 1.16		
4. The staff encouraged me to try new ways of coping (such as breathing techniques).	2.42 ± 1.10		
5. The staff realized the pain I was in	3.45 ± 1.17		1
6. The staff encouraged me not to fight what my body was doing	3.20 ± 1.15		2
7. I felt like the staff had their own agenda*	2.40 ± 1.05		
8. I felt like the staff tried to move things along for their own convenience*	2.48 ± 1.13		
9. I was given time to ask questions	2.01 ± 1.24	2	
10. The staff helped me to try different positions	1.45 ± 0.89	1	
11. The staff stopped doing something if I asked them to stop	2.18 ± 1.03	3	
12. The staff dismissed things I said to them	2.26 ± 1.13		

* Items = Reversed scored

Ford, Ayers & Wright (2009); Measurement of Maternal Perceptions of Support and Control in Birth (SCIB)

### The difference in demographic-obstetric characteristics and Women’s perception support and control during childbirth

#### Perception of internal control

Higher levels in perception of internal control was significantly associated with participants aged 25 years and older (*rho* = .20; *p* = .004), and vaginal delivery (median = 2.20, *U* = 374.0, *p* = .004).

#### Perception of external control

Higher levels in perception of external control was significantly associated with participants aged 25 years and older (*rho* = .17; *p* = .02), vaginal delivery (median = 1.91, *U* = 419.0, *p* = <.001), and higher parity (median = 1.91, *U* = 2661.0, *p* = .001); and a low level in perception of external control significantly associated with women who delivered in Western Region (median = 1.73, *U* = 2721.0, *p* = .001), and those who had primary or higher education (median = 1.73, *U* = 3647.0, *p* = .001).

#### Perception of support

There was a statistically significant higher level in perception of support among married women (median = 2.58, *U* = 310, *p* = .05), women who had a vaginal delivery (median = 2.58, *U* = 326.50, *p* = .002), participants aged 25 years and older (*rho* = .18, *p* = .01), higher parity (median = 2.58, *U =* 2960.50, *p* = .01) and those who had a birth plan (median = 2.67, *U* = 3649.0, *p* = .001). 2.58). (Additional file 3: Table S3), shows the difference in demographic-obstetric factors and women’s perception of maternal support and control during childbirth.

### Predictors of Women’s perception of support and control during childbirth

#### Perception of internal control

Mode of delivery (*p* = .01) and age (*p* < .001) were significant predictors for women’s perception of internal control during childbirth, *F*_(3,196)_ = 6.74, *p* < .001, and 8% of the variance in perception of internal control was explained by the model. Women with aged 25 years and older and those who had a vaginal delivery were more likely to have a higher level in the perception of internal control during childbirth.

#### Perception of external control

Women’s perception of external control was significantly predicted by mode of delivery (*p* = .04), place of delivery (*p* < .001), educational status (*p* = .02) and perception of support (*p* < .001), (*F*_(6, 182)_ = 10.12; *p* < .001), indicating that 27% of the variance in perception of external control was explained by the model. Women who had a vaginal delivery were more likely to have a higher level in the perception of external control. Perception of external control was more likely to be high as the perception of support increased during childbirth, and women who had primary or higher education, and those who delivered in Western Region were less likely to have higher levels in the perception of external control during childbirth.

#### Perception of support

Birth plan (*p* = .001), mode of delivery (*p* = .04), and perception external control (*p* < .001) significantly predicted women’s perception of support, *F*_(7, 192)_ = 8.75; *p* < .001, and 25% of variance in perception of support was explained by the model. Women who had a vaginal delivery, those who had a birth plan, and those with a higher level in the perception of external control had a high perception of support during childbirth. (Additional file 4: Table S4), shows related factors predicting women’s perception of support and control during childbirth.

## Discussion

The purpose of this study was to explore women’s perception of support and control during childbirth in the Gambia. According to Ford et al. (2009), perception of control is on two folds namely; internal control which measured women’s experience of labour in terms of thoughts, emotions, behaviours, pain and physical functioning during labour and delivery. External control assessed women’s control over events controlled by external forces such as access to information, the birth environment, decision making and procedures, and birth outcomes. Perception of support assessed women’s perception of supportive care from skilled birth attendants, including staff attitude, encouragement, coaching, empathy, listening and providing information [22]. The findings, therefore, help to answer the following research questions.

### What is the women’s perception of support and control during childbirth in the Gambia?

As evident in this study, the overall perceptions of control and support were low. Among the three subscales, external control 1.85 (SD ± 0.43) recorded the least perception, which implies that women had the lowest perceived control over external factors such as accessing information and decision making during childbirth. The finding is in line with a similar study conducted in the United Kingdom, which revealed a lower level of perception in external control than the perception of internal control among women during childbirth [22]. Internal control is when women trust in their capability to control events through their own efforts [20] and their experience of labor in terms of their thoughts, emotions, behaviors, pain and physical functioning during labor and delivery [22]. Therefore, this finding could be related to participants’ belief in culture, because childbirth in The Gambia is viewed as a transition to adulthood and an experience to showcase womanhood [25], therefore, women are expected to endure labor pains and behave positively during the process.

Factors with the lowest perception of internal control included “the inability to control reactions to pain”, “being mentally calm during labor” and “the pain being too great to gain control”. This finding supports Green and Baston [17] in which labor pain was regarded as the factor influencing poor control during childbirth. Severe pain during childbirth increases the production of adrenaline, which may interfere with the progress of labor. Therefore, non-pharmacological pain management such as massage, which is the only intrapartum pain management intervention available in The Gambia, should be provided to women.

External factors such as “deciding when they receive information”, “inability to influence which procedures were carried out” and “inability to gain control over what information was given to them” had the lowest scores. This implies that women had less autonomy as regards to information sharing and involvement in decision-making during childbirth, and this is a form of mistreatment and loss of dignity [11]. Women’s involvement in decision making, makes them feel dignified and respected during labor. [17]. Effective communication which is both woman-initiated and provider-initiated is a main element of gaining external control [8].

Though women’s perception of support was low, it was higher than the perception of both internal and external control in this study. This corroborates with the findings of a similar study conducted in the United Kingdom [22]. One of the factors with the lowest perception of support included “women not helped to try different positions during birth”. Empowering and encouraging women to change positions during the first and second stage of labour is an integral part of quality intrapartum care [26]. Maternal positioning as preferred by women during birth act as a coping mechanism for pain and promote comfort [26], and utilizing positions such as the upright position during the second stage of labor, minimizes obstetric complications and interventions, shorten the duration of labor and reduces the feeling of pain [27]. In The Gambia, the supine/lithotomy position is the most widely used position during the second stage of labor in the health facilities across the country, this is also evident in Malawi [28]. Lack of knowledge and competence in conducting deliveries using positions other than the supine position could be one of the reasons for women not helped to try different positions in this study. Positioning during delivery is not captured in The Gambia Maternity Care Guideline and Service Delivery Standards [29], therefore, it should be incorporated, and training on the use of different positions during delivery should be provided to skilled birth attendants (midwives), to improve competence. The skills should also be incorporated in all the midwifery curricula in The Gambia.

Women not given the time to ask questions was also one of the factors with the lowest perception of support. This implies that the interaction between skilled birth attendants and women was poor. Effective client-centered communication by healthcare providers during childbirth is regarded as an enhancer for respectful and dignified childbirth [30]. The Reproductive and Child Health Unit and the midwifery institutions in The Gambia should put more emphasis on improving the interpersonal skills of skilled birth attendants to enhance effective maternity care.

Women’s inability to stop skilled birth attendants in doing something to them they don’t like is a form of non-consent care, which is a violation of women’s right and an intentional act of disrespect and abuse during childbirth [30, 31]. Therefore, women should be empowered to make their own decisions about the care provided, which must be respected as it is crucial in promoting control during childbirth.

### What are the related factors influencing women’s perception of support and control during childbirth in the Gambia?

Women with aged 25 years and older were more likely to have an increase in perception of internal control. The finding agrees with Weisman et al. [20], in which the perception of internal control was negatively associated with younger maternal age. In the same vein, women who had a normal vaginal delivery were more likely to have a higher level in the perception of internal control and perception of external control as compared to those who had instrumental delivery. This finding is consistent with the findings of Ford et al. and Bertucci et al. [22, 32], which stated that birth interventions such as instrumental deliveries may influence the perception of control among women during childbirth.

Education is one of the social determinants of health, and research has shown that the higher the patients’ level of education, the higher the expectation of care provided [33]. In this study, higher levels of education were associated with a decrease in perception of external control, which shows that educated women had a lower expectation about the care received and this may be due to their ability to express their wishes. Due to the low literacy rate among women compared to their male counterparts in The Gambia [34], this study supports the need for the government to step up efforts in promoting female education.

Women who delivered in Western Region were less likely to have a higher level in the perception of external control. Though the reason for this finding is not clear, the high number of deliveries [15] and the low provider-client ratio [35] in health facilities may be responsible. However, further research is needed to understand this phenomenon.

Perception of support was positively associated with the perception of external control. Supportive care from health care providers is an important factor that promotes control during childbirth [8]. Therefore, supportive care from skilled birth attendants should be provided to women in order to enhance external control during childbirth.

Regarding the perception of support, the findings further demonstrated that mode of delivery, the perception of external control and birth plan were the significant predictors. Women who had a birth plan and those who had a vaginal delivery were more likely to have higher levels in the perception of support. Effective implementation of the birth preparedness and complication readiness strategies should be strengthened in antenatal care services across the country, as the birth plan has been identified as a factor that enhances the feeling of support during childbirth [8]. Individualized care in which care is provided based on women’s needs should be strengthened to improve quality care.

The study revealed a positive association between perception of external control and perception support. Therefore, for women to feel supported, external factors that affect women’s autonomy during childbirth such as poor client-provider communication and women’s inability to make decisions about their own care should be eliminated.

The study findings therein will enhance the formulation and implementation of effective tailor-made dignified intrapartum care policies that will have a positive impact on women’s experience of childbirth, so as to improve the utilization of facility-based intrapartum care service in The Gambia.

Concerning the limitations of this study, a convenience sampling was the method employed in selecting participants in this study. This sampling method is liable to selection bias. The three health facilities identified for the study may differ in the implementation of intrapartum care protocols, and this may have an impact on the results of this study.

## Conclusion

Ensuring universal access to reproductive health for all women is one of the components of the Sustainable Development Goals, and is the fundamental desired goal for countries worldwide. Therefore, The Gambia as a developing country with high maternal and infant mortality rates needs to double up attempts to improving the utilization of reproductive health services nationwide. It is therefore critical for The Gambia and other developing countries facing the challenges of moving towards dignified birth, to create an environment within all maternity units that will provide supportive care and promote feelings of control during childbirth, so as to enhance positive decisions about the utilization of maternity care services in future pregnancies.

This study indicated that participants had the lowest perception in pain control, involvement in decision making regarding the care provided, information sharing and utilising different position during birth. Age, mode of delivery, educational status, place of delivery and birth plan significantly predicted participants’ perceptions of support and control during childbirth.

From these findings, it is clear that there is a need for the Reproductive and Child Health Unit (RCH) and the Nurses and Midwives Council of The Gambia to strengthen efforts in ensuring that women are given the right support and control during childbirth. This can be achieved through effective supervision and training of skilled birth attendants on non-pharmacological pain management, effective client-provider communication during the intrapartum period, and promote women’s involvement in decision making. The findings also support the need for all midwifery curricula to incorporate the training of skilled birth attendants (midwives) on the use of different positions to conduct deliveries, as they are equally important in promoting women feeling a sense of control and support during childbirth.

## Additional files

Additional file 1: Demographic-obstetric information questionnaire. (DOCX 28 kb)

Additional file 2: The Support and Control in Birth (SCIB) scale - English version. (PDF 43 kb)

Additional file 3: **Table S3.** Showing the difference in demographic-obstetric characteristics and women’s perception support and control during childbirth. (DOCX 15 kb)

Additional file 4: **Table S4.** Showing predictors of women’s perception of support and control during childbirth. (DOCX 13 kb)

## Abbreviations

GBOS: Gambia Bureau of Statistics
ICPD: International Conference on Population and Development
MOHSW: Ministry of Health and Social Welfare
RCH: Reproductive and Child Health
SCIB: Support and Control in Birth Scale
TBA: Traditional Birth Attendants
UNDP: United Nation Development Programme
WHO: World Health Organisation
