# Supplementary material for: Women’s perception of support and control during childbirth in The Gambia, a quantitative study on dignified facility-based intrapartum care
Source: BMC Pregnancy Childbirth. 2018 Oct 23;18:413. doi: 10.1186/s12884-018-2025-5 (PMC6199796; doi:10.1186/s12884-018-2025-5)
Supplement: Supplementary file 1 — Demographic-obstetric information questionnaire. (DOCX 28 kb) [file 12884_2018_2025_MOESM1_ESM.docx]

**The Questionnaire**

**DEMOGRAPHIC-OBSTETRIC INFORMATION SHEET**

**Serial number: ….. Health region code:…. Participant code:…..**

**Date: …../...../…..**

**Section A: DEMOGRAPHY**

1. What is your age?......................years
2. What is your level of education
3. Tertiary level
4. Secondary level
5. Primary level
6. None
7. Marital status…………………
8. Married
9. Single
10. What is your ethnicity
11. Jola
12. Fula
13. Wollof
14. Mandinka
15. Other specify………………………….

**Section B: Obstetric Factors**

7 How many children did you have in total including the deaths?

1. 1
2. 2
3. 3
4. 4

8 How many times have attended routine antenatal clinic for this baby?

1. none
2. 1
3. 2
4. 3
5. 4 or more

9 Did you have any birth plan during pregnancy with midwife for this baby?

1. No
2. Yes

10 By what method did you deliver this baby?

1. Normal vaginal delivery
2. Assisted vaginal delivery
3. Cesarean Section

**Section C: Support and Control in Birth Scale (SCIB)**

This questionnaire asks about your experience of the labour and birth of your baby. Please indicate you level of agreement with the following statements.

| **No.** | **Scoring**  **1= completely disagree 2= disagree**  **3= neither agree nor disagree 4= agree**  **5= completely disagree**  **^a^ Items should be reversed scored.** | **Scores** | | | | | **Official use** |
| --- | --- | --- | --- | --- | --- | --- | --- |
|  | **INTERNAL CONTROL** | **1** | **2** | **3** | **4** | **5** |  |
| N 1 | The pain was too great for me to gain control over it **^a^**. |  |  |  |  |  |  |
| N2 | I was overcome by the pain |  |  |  |  |  |  |
| N3 | I was able to control my reaction to the pain |  |  |  |  |  |  |
| N4 | I was mentally calm |  |  |  |  |  |  |
| N5 | I was in control of my emotions |  |  |  |  |  |  |
| N6 | I felt my body was on a mission that I could not control **^a^** |  |  |  |  |  |  |
| N7 | Negative feelings overwhelmed me **^a^** |  |  |  |  |  |  |
| N8 | I gained control by working with my body |  |  |  |  |  |  |
| N9 | I could control the sounds I was making |  |  |  |  |  |  |
| N10 | I behaved in a way not like myself **^a^** |  |  |  |  |  |  |
|  | **EXTERNAL CONTROL** |  |  |  |  |  |  |
| E 1 | I had control over when procedures happened |  |  |  |  |  |  |
| E2 | I could influence which procedures were carried out. |  |  |  |  |  |  |
| E3 | I decided whether procedures were carried out or not. |  |  |  |  |  |  |
| E 4 | The people in the room took control **^a^** |  |  |  |  |  |  |
| E5 | I had control over the decisions that were carried out or not. |  |  |  |  |  |  |
| E6 | I could get up and move around as I much as I wanted. |  |  |  |  |  |  |
| E7 | People coming in and out of the room was beyond my control **^a^**. |  |  |  |  |  |  |
| E8 | I chose whether I was given information or not |  |  |  |  |  |  |
| E9 | I could decide when I received information |  |  |  |  |  |  |
| E10 | I had control over what information I was given |  |  |  |  |  |  |
| E11 | I felt I had control over the way my baby was finally born |  |  |  |  |  |  |
|  | **SUPPORT** |  |  |  |  |  |  |
| S1 | The staff helped me find energy to continue when I wanted to give up |  |  |  |  |  |  |
| S2 | The staff seemed to know instinctively what I wanted or needed. |  |  |  |  |  |  |
| S3 | The staff went out of their way to try to keep me comfortable. |  |  |  |  |  |  |
| S4 | The staff encouraged me to try new ways of coping  ( such as breathing techniques) |  |  |  |  |  |  |
| S5 | The staff realized the pain I was in |  |  |  |  |  |  |
| S6 | The staff encouraged me not to fight what my body was doing. |  |  |  |  |  |  |
| S7 | I felt the staff had their own agenda **^a^** |  |  |  |  |  |  |
| S8 | I felt like the staff tried to move things along for their own convenience **^a^** |  |  |  |  |  |  |
| S9 | I was given time to ask questions |  |  |  |  |  |  |
| S10 | The staff helped me to try different positions |  |  |  |  |  |  |
| S11 | The staff stopped doing something if I asked them to stop. |  |  |  |  |  |  |
| S12 | The staff dismissed things I said to them. |  |  |  |  |  |  |
