# Supplementary material for: Women’s perception of support and control during childbirth in The Gambia, a quantitative study on dignified facility-based intrapartum care
Source: BMC Pregnancy Childbirth. 2018 Oct 23;18:413. doi: 10.1186/s12884-018-2025-5 (PMC6199796; doi:10.1186/s12884-018-2025-5)
Supplement: Supplementary file 2 — The Support and Control in Birth (SCIB) scale - English version. (PDF 43 kb) [file 12884_2018_2025_MOESM2_ESM.pdf]

Support & Control in Birth (SCIB)

This questionnaire asks about your experience of the labour and birth of your baby. If you had a planned caesarean, please answer the questions as best you can for how your experience was.

What kind of control did you have during labour and birth?
If a question is not relevant to your experience, please mark the box "neither".

		Agree completely	Agree slightly	Neither	Disagree slightly	Disagree completely
1	I had control over when procedures happened	☐	☐	☐	☐	☐
2	I could influence which procedures were carried out	☐	☐	☐	☐	☐
3	I decided whether most procedures were carried out or not	☐	☐	☐	☐	☐
4	I had control over the decisions that were made	☐	☐	☐	☐	☐
5	The people in the room took control	☐	☐	☐	☐	☐
6	People coming in and out of the room was beyond my control	☐	☐	☐	☐	☐
7	I could get up and move around as much as I wanted	☐	☐	☐	☐	☐
8	I chose whether I was given information or not	☐	☐	☐	☐	☐
9	I could decide when I received information	☐	☐	☐	☐	☐
10	I had control over what information I was given	☐	☐	☐	☐	☐
11	I felt I had control over the way my baby was finally born	☐	☐	☐	☐	☐
12	The pain was too great for me to gain control over it	☐	☐	☐	☐	☐
13	I was overcome by the pain	☐	☐	☐	☐	☐
14	I was mentally calm	☐	☐	☐	☐	☐
15	I was able to control my reactions to the pain	☐	☐	☐	☐	☐
16	I was in control of my emotions	☐	☐	☐	☐	☐
17	I felt my body was on a mission that I could not control	☐	☐	☐	☐	☐
18	Negative feelings overwhelmed me	☐	☐	☐	☐	☐
19	I gained control by working with my body	☐	☐	☐	☐	☐
20	I behaved in a way not like myself	☐	☐	☐	☐	☐
21	I could control the sounds I was making	☐	☐	☐	☐	☐

© Ford E, Ayers S & Wright DB (2009) Measurement of maternal perceptions of support and control on birth (SCIB). Journal of Women's Health, 18(2) 245-252.

What kind of support did you receive from healthcare staff during labour and birth?

		Agree completely	Agree slightly	Neither	Disagree slightly	Disagree completely
22	The staff helped me find energy to continue when I wanted to give up	☐	☐	☐	☐	☐
23	The staff knew instinctively what I wanted or needed	☐	☐	☐	☐	☐
24	The staff went out of their way to try to keep me comfortable	☐	☐	☐	☐	☐
25	The staff encouraged me to try new ways of coping	☐	☐	☐	☐	☐
26	The staff encouraged me not to fight against what my body was doing	☐	☐	☐	☐	☐
27	The staff realized the pain I was in	☐	☐	☐	☐	☐
28	I felt the staff had their own agenda	☐	☐	☐	☐	☐
29	I was given time to ask questions	☐	☐	☐	☐	☐
30	I felt like the staff tried to move things along for their own convenience	☐	☐	☐	☐	☐
31	The staff helped me to try different positions	☐	☐	☐	☐	☐
32	The staff stopped doing something if I asked them to stop	☐	☐	☐	☐	☐
33	The staff dismissed things I said to them	☐	☐	☐	☐	☐

© Ford E, Ayers S & Wright DB (2009) Measurement of maternal perceptions of support and control on birth (SCIB). Journal of Women's Health, 18(2) 245-252.
