# Supplementary material for: Women’s perception of support and control during childbirth in The Gambia, a quantitative study on dignified facility-based intrapartum care
Source: BMC Pregnancy Childbirth. 2018 Oct 23;18:413. doi: 10.1186/s12884-018-2025-5 (PMC6199796; doi:10.1186/s12884-018-2025-5)
Supplement: Supplementary file 3 — Table S3. Showing the difference in demographic-obstetric characteristics and women’s perception support and control during childbirth. (DOCX 15 kb) [file 12884_2018_2025_MOESM3_ESM.docx]

**Additional file 3: Table S3 The Difference in Women's Demographic-obstetric Characteristics and Perception Support and Control during Childbirth (N = 200)**

| **Variables** |  | **Internal** | **Control** |  | **External** | **Control** |  |  | **Support** |  |
| --- | --- | --- | --- | --- | --- | --- | --- | --- | --- | --- |
|  | **N** | **Median** | ***rho/U/H*** | ***p*-value** | **Median** | ***rho/U/H*** | ***p*-value** | **Median** | **rho/U/H** | **p-value** |
| Age |  |  | .20^a^ | .004 |  | .17^a^ | .02 |  | .18^a^ | .01 |
| Marital status |  |  | 357.0^b^ | .11 |  | 277.50^b^ | .03 |  | 310.0^b^ | .05 |
| Not married | 6 | 2.00 |  |  | 1.41 |  |  | 1.75 |  |  |
| Married | 194 | 2.20 |  |  | 1.82 |  |  | 2.58 |  |  |
| Educational status |  |  | 4190.0^b^ | .06 |  | 3647.0^b^ | .001 |  | 4700.50^b^ | .54 |
| No formal education | 110 | 2.20 |  |  | 2.00 |  |  | 2.50 |  |  |
| Primary or higher | 90 | 2.15 |  |  | 1.73 |  |  | 2.67 |  |  |
| Ethnicity |  |  | 1.60^c^ | .45 |  | 2.43^c^ | .30 |  | 4.70^c^ | .10 |
| Mandinka | 80 | 2.15 |  |  | 1.73 |  |  | 2.63 |  |  |
| Fula | 47 | 2.20 |  |  | 2.00 |  |  | 2.58 |  |  |
| Others | 73 | 2.20 |  |  | 1.82 |  |  | 2.33 |  |  |
| Antenatal attendance |  |  | 4947.0^b^ | .90 |  | 4868.0^b^ | .75 |  | 4615.50^b^ | .35 |
| < 4 visits | 101 | 2.20 |  |  | 1.91 |  |  | 2.58 |  |  |
| ≥ 4 visits | 99 | 2.20 |  |  | 1.82 |  |  | 2.50 |  |  |
| Mode of delivery |  |  | 374.0^b^ | .004 |  | 419.0^b^ | <.001 |  | 326.50^b^ | .002 |
| Vaginal delivery | 191 | 2.20 |  |  | 1.91 |  |  | 1.50 |  |  |
| Instrumental delivery | 9 | 1.90 |  |  | 1.27 |  |  | 2.58 |  |  |
| Place of delivery |  |  | 3693.50^b^ | .42 |  | 2721.0^b^ | .001 |  | 3791.0^b^ | .59 |
| Western Region | 145 | 2.20 |  |  | 1.73 |  |  |  |  |  |
| Lower River Region | 55 | 2.10 |  |  | 2.00 |  |  |  |  |  |
| Parity |  |  | 3291.0^b^ | .12 |  | 2661.0^b^ | .001 |  | 2960.50^b^ | .01 |
| Nulliparous | 52 | 2.10 |  |  | 1.64 |  |  | 2.29 |  |  |
| Multiparous | 148 | 2.20 |  |  | 1.91 |  |  | 2.58 |  |  |
| Birth plan |  |  | 4329.50^b^ | .13 |  | 4864.50^b^ | .85 |  | 3649.0^b^ | .001 |
| No | 111 | 2.20 |  |  | 1.82 |  |  | 2.42 |  |  |
| Yes | 89 | 2.10 |  |  | 1.91 |  |  | 2.67 |  |  |

**a = Spearman’s *rho* correlation; b = Mann-Whitney *U* test; c = Kruskal Wallis *H* test**
