# Supplementary material for: Women’s perception of support and control during childbirth in The Gambia, a quantitative study on dignified facility-based intrapartum care
Source: BMC Pregnancy Childbirth. 2018 Oct 23;18:413. doi: 10.1186/s12884-018-2025-5 (PMC6199796; doi:10.1186/s12884-018-2025-5)
Supplement: Supplementary file 4 — Table S4. Showing predictors of women’s perception of support and control during childbirth. (DOCX 13 kb) [file 12884_2018_2025_MOESM4_ESM.docx]

**Additional file 4: Table S4 Predictors of Women's Perception of Support and Control during Childbirth (N = 200)**

| **Variables** |  |  | **Perceptions** |  |  |  |
| --- | --- | --- | --- | --- | --- | --- |
|  | **Internal Control** | **p-value** | **External Control** | **p-value** | **Support** | **p-value** |
|  | **B (SE)** |  | **B (SE)** |  | **B (SE)** |  |
| Constant | 1.54 (.25) | <.001 | .83 (.21) | <.001 | .75 (.31) | .02 |
| < 25 yrs vs, ≥ 25 yrs | .33 (.09) | <.001 | .02 (.06) | .79 | .10 (.09) | .28 |
| Instrumental delivery vs. vaginal delivery | .55 (.22) | .01 | .28 (.14) | .04 | .42 (.20) | .04 |
| Nulliparous vs. multiparous |  |  | .07 (.07) | .31 |  |  |
| Western Region vs. Lower River Region |  |  | -.22 (.06) | <.001 |  |  |
| No formal education vs. Primary or higher |  |  | -.14 (.06) | .02 |  |  |
| Married vs. not married |  |  | .09 (.17) | .59 | .18 (.24) | .46 |
| Birth plan vs. no birth plan |  |  |  |  | .27 (.08) | .001 |
| Perception of internal control |  |  |  |  | .03 (.06) | .70 |
| Perception of external control |  |  |  |  | .48 (.10) | <.001 |
| Perception of Support | .06 (.08) | .42 | .24 (.05) | <.001 |  |  |

*Perception of Internal control: F (3, 196) = 6.74; p<.001; R^2^ = .09; Adjusted R^2^ = .08*

*Perception of external control: F (7, 192) = 10.12; p < .001; R^2^ = .27; Adjusted R^2^ = .24*

*Perception of support: F (7, 192) = 8.75; p < .001; R^2^ = .24; Adjusted R^2^ = .21*
